# Comparison of gait parameters between patients with chronic stroke at different ambulation levels and healthy adults: a prospective observational study

**DOI:** 10.1186/s13102-025-01444-4

**Published:** 2025-12-01

**Authors:** Se-Young Bak, Eun-Hye Chung, Heegoo Kim, Seyoung Shin, HyeongMin Jeon, MinYoung Kim

**Affiliations:** 1 Department of Rehabilitation Medicine, CHA Bundang Medical Center, CHA University School of Medicine, Seongnam-si, Republic of Korea; 2 Digital Therapeutics Research Team, CHA Bundang Medical Center, Seongnam-si, Republic of Korea; 3 Department of Medicine, CHA University, Seongnam-si, Republic of Korea

**Keywords:** Dependent ambulation, Gait, Kinematics, Kinetics, Stroke

## Abstract

**Background:**

The mechanisms underlying gait function recovery in stroke remain uncertain. Biomechanical gait analysis has emerged as a promising approach to address this gap, offering essential information for developing tailored gait rehabilitation strategies in patients with stroke. However, few studies have investigated the gait biomechanics of dependent stroke ambulators, particularly for patients classified as level 3 in the Functional Ambulation Category (FAC), which refers to the ability to walk on a level surface under supervision.

**Methods:**

This prospective observational study recruited twelve patients with chronic stroke with an onset duration of more than six months, along with six healthy adults. The patients with stroke were grouped into FAC level 3 (N.=6) or FAC level 4 (N.=6) based on their level of independence. All participants performed level walking along a 7-meter walkway while three-dimensional motion capture was used to assess gait biomechanics. Seven functional assessments, including the Berg Balance Scale and Trunk Impairment Scale, were also conducted in patients with stroke. The Kruskal–Wallis test and one-way analysis of variance were used to compare gait parameters among groups, followed by Mann–Whitney and independent t-tests for post-hoc analyses.

**Results:**

Both stroke groups showed significant differences in biomechanical parameters compared to the healthy group (*p* < 0.05). Compared to the FAC 4 group, the FAC 3 group exhibited significantly lower peak posterior ground reaction force on the affected side (*p* = 0.002); reduced hip range of motion (*p* = 0.047) and peak hip flexion moment (*p* = 0.044), and maximum knee flexion angle on the unaffected side (*p* = 0.026). Compared to the healthy group, the FAC 3 group demonstrated significantly reduced ankle range of motion on the affected side (*p* = 0.021), and lower maximum hip extension angle (*p* = 0.011), lower peak hip extension moment (*p* = 0.031) and peak ankle dorsiflexion moment lower maximum hip extension angle (*p* = 0.004) on the unaffected side, while no differences were observed between the FAC 4 and healthy groups (*p* > 0.05).

**Conclusions:**

Significant differences in biomechanical parameters, particularly those related to eccentric contraction in the proximal joints of the unaffected side, were observed between FAC 3 and FAC 4 groups. These disparities highlight the need for tailored gait rehabilitation strategies based on ambulation level.

**Trial registration:**

Clinical Trial No. NCT05908994, Date of registration: 23/05/2023.

**Supplementary Information:**

The online version contains supplementary material available at 10.1186/s13102-025-01444-4.

## Introduction

Stroke survivors frequently present with hemiplegia, which contributes to persistent gait impairments, including reduced walking speed, increased asymmetry, and compensatory movements [1–3]. For example, a previous study [4] reported that post-stroke gait dysfunction commonly involves spatio-temporal, kinematic, kinetic, and electromyographic deviations compared to healthy peers. Given that walking ability remains a key determinant of mobility and social participation, restoring functional gait remains a central goal of rehabilitation [5, 6]. Despite this, the mechanisms driving gait recovery after stroke, particularly how biomechanics evolve across levels of ambulatory independence, remain incompletely characterized. While studies have described just specific pattern of deviations without considering severity of impairment [7], few have stratified findings by ambulation categories, such as the Functional Ambulation Category (FAC).

Traditional rehabilitative gait training for stroke have largely emphasized strengthening concentric contractions of the hip and knee musculature [8–11]. In contrast, emerging evidence suggests that eccentric contraction training may yield greater neuromuscular activation, power gains and walking speed improvements in post-stroke patients [12, 13]. However, while promising, the mechanisms underpinning eccentric-based training and detailed biomechanical changes during gait remain under-explored [1, 10]. Moreover, the literature lacks detailed three-dimensional biomechanical analyses linking ambulation level (e.g., FAC 3 vs. FAC 4) to joint kinetics, kinematics and ground reaction forces. Leveraging three-dimensional motion capture to characterize these biomechanical differences may therefore offer valuable insights for tailoring rehabilitation strategies [14–18].

The scope of gait ability spans a continuum from assisted walking to independent locomotion on complex terrains such as stairs or uneven ground [19, 20]. However, most previous biomechanical studies [14–18] have primarily investigated stroke survivors who are already capable of independent ambulation, often treating them as a homogeneous group without stratifying their functional levels. This approach overlooks the considerable variability in gait control, compensatory mechanisms, and motor coordination that occurs across different stages of ambulatory recovery. In clinical rehabilitation, FAC is widely recognized as a valid and reliable tool for classifying gait ability according to the level of physical assistance required during ambulation [21]. Despite its clinical utility, the use of FAC as a stratification framework in biomechanical research remains limited. Notably, a recent study of our study team [22] examining stroke survivors at FAC levels 4 and 5 reported significant differences in hip and ankle joint power generation between the groups. These findings highlight that meaningful biomechanical distinctions exist even among independent ambulators, underscoring the importance and necessity of classifying stroke patients by FAC levels when analyzing gait biomechanics.

However, to date, no studies have examined gait biomechanics in stroke patients classified as FAC 3, representing individuals who can ambulate only under close supervision on level ground. Clinically, the transition between FAC 3 and FAC 4 marks a critical threshold between dependent and independent walking, reflecting substantial differences in balance control, motor coordination, and compensatory gait strategies. Accordingly, stratifying patients by FAC level, particularly between levels 3 and 4 can provide essential biomechanical insights and guide the design of tailored rehabilitation programs for each ambulatory recovery stage. Therefore, this study aimed to compare lower-extremity kinematic and kinetic parameters among stroke survivors with FAC 3 or FAC 4 and healthy adults. We hypothesized that biomechanical parameter differences across FAC levels would provide mechanistic insights for developing individualized gait rehabilitation strategies.

## Materials and methods

### Participants

The required sample size was calculated through the G-power program [23, 24]. This study aimed to compare the differences according to FAC grades, hence; the authors anticipated setting one effect and using one-way ANOVA. Considering the rare number of patients with FAC 3, the authors set effect size of 0.7, larger than 0.4 which is generally used, α of 0.1, and power of 0.75. Consequently, sample size was finally decided as 18. Hence, this prospective and observational study recruited twelve patients with chronic stroke (more than six months after onset) and six healthy adults. Patients with stroke were recruited from the outpatient Department of Rehabilitation Medicine at CHA Bundang Medical Center from August 2023 to August 2024. Healthy adults were recruited through participation notices during the same period as the patients with stroke. The healthy volunteers were pre-screened based on the medical examination by rehabilitation doctors and medical history. Patients with stroke were classified into two groups according to the FAC: level 3, indicating dependent ambulation requiring supervision, and level 4, indicating independent ambulation on even surfaces. The classification was conducted by a single researcher (Se-Young Bak, licensed physical therapist). To minimize potential bias during evaluation, the researcher was blinded to the participants’ pathological and clinical histories. The demographic and pathological characteristics of the participants are summarized in Table 1 and Supplementary Table S1, respectively. All participants were informed of the purpose of the study and voluntarily provided written informed consent prior to participation. Exclusion criteria included the presence of systemic infection symptoms, cognitive impairment (including mild cognitive impairment), a Mini-Mental State Examination (MMSE) score of < 10, unstable internal medical conditions (e.g., cardiovascular, gastrointestinal, or respiratory instability), or any situation deemed inappropriate for participation by the researchers, such as temporary deterioration of the participant’s health status or excessive fatigue. In accordance with the declaration of Helsinki, this study was approved by the CHA Bundang Medical Center IRB review committee (Clinical trial No. NCT05908994, registration date: 23/05/2023; IRB 2021-05−026).

**Table 1:** Participant demographic and pathological characteristics

Characteristics	FAC 3 group (*N*.=6)	FAC 4 group (*N*.=6)	Healthy group (*N*.=6)	*p*-values	95% Confidence Interval	
					Lower	Upper
Gender (male/female)	4/2	4/2	4/2	1.000	-	-
Age (years)	66.33 ± 5.24	68.33 ± 9.95	65.17 ± 3.97	0.728	-	-
Height (cm)	163.33 ± 8.98	162.50 ± 7.04	163.02 ± 11.15	0.988	-	-
Weight (kg)	62.58 ± 10.13	61.52 ± 8.34	67.25 ± 14.98	0.663	-	-
Onset duration (days)	1220.67± 863.66	1316.00± 596.80	-	0.828	−1050.268	859.601
Unaffected side (right/left)	3/3	3/3	-	1.000	−0.705	−0.705

Continuous values are presented as mean±standard deviation. Gender, age, height, and weight were analyzed by ANOVA test, while onset duration and affected side were analyzed by independent t-test. For analysis of gender data, male was coded as 1 and female was coded as 2. For analysis of unaffected side data, right was coded as 1 and left was coded as 2

### Equipment and environment

**Fig. 1:**
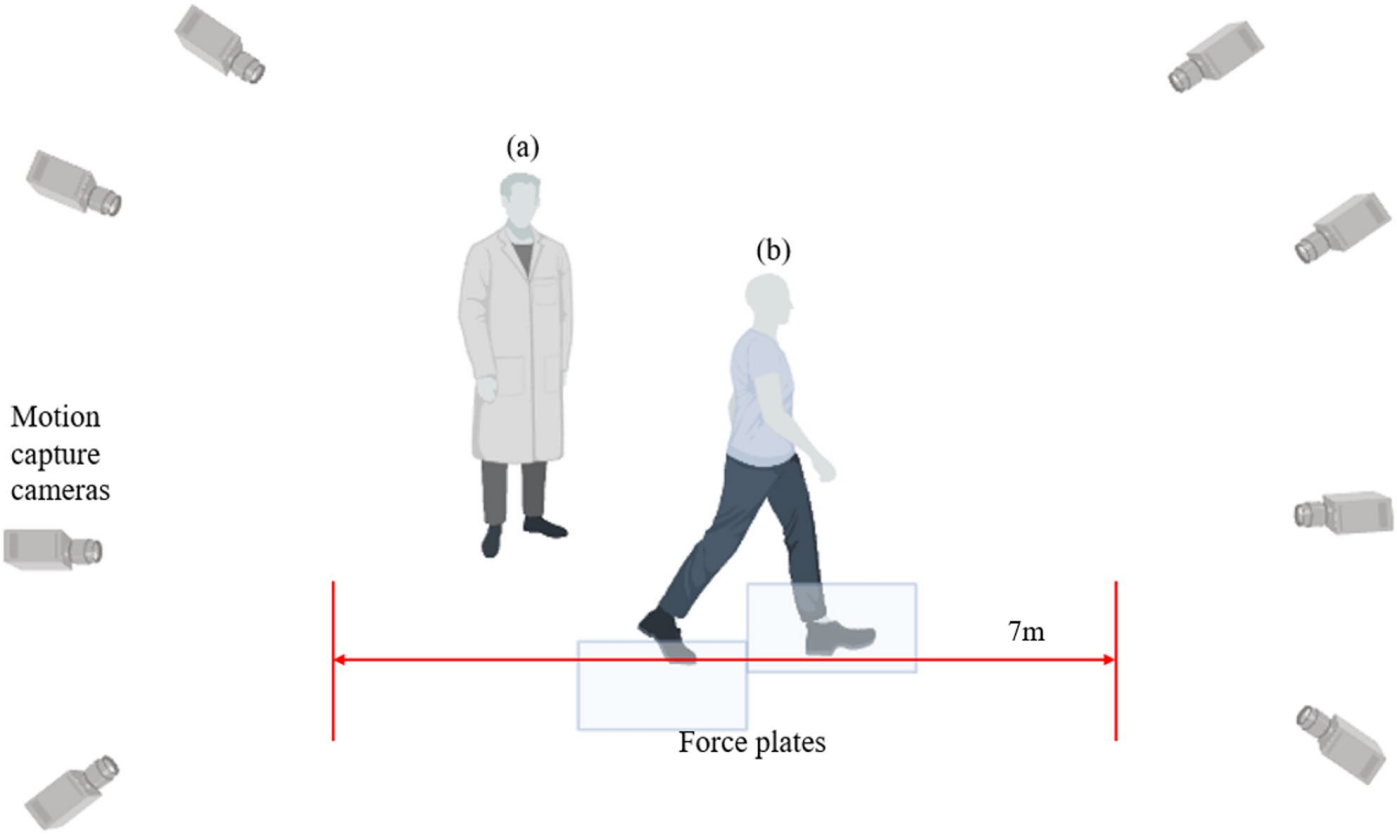
Experimental environment for gait analysis. (**a)** a researcher, (**b**) a participant

Kinematic data were obtained at 100 Hz using eight 3D motion capture cameras (Miqus Hybrid, Qualisys, Sweden), and kinetic data were obtained at 1000 Hz from two force plates (9260AA6, Kistler, Switzerland) during gait. The eight cameras were installed on all sides to capture the participants clearly, and the two force plates were located at the center of the seven-meter gait path. The position of the force plates was adjusted so that each participant could naturally step with the entire foot on each plate during the stance phase after practice trials. The gait path and the experimental environment are shown in Fig. 1.

### Experimental protocol

**Fig. 2:**
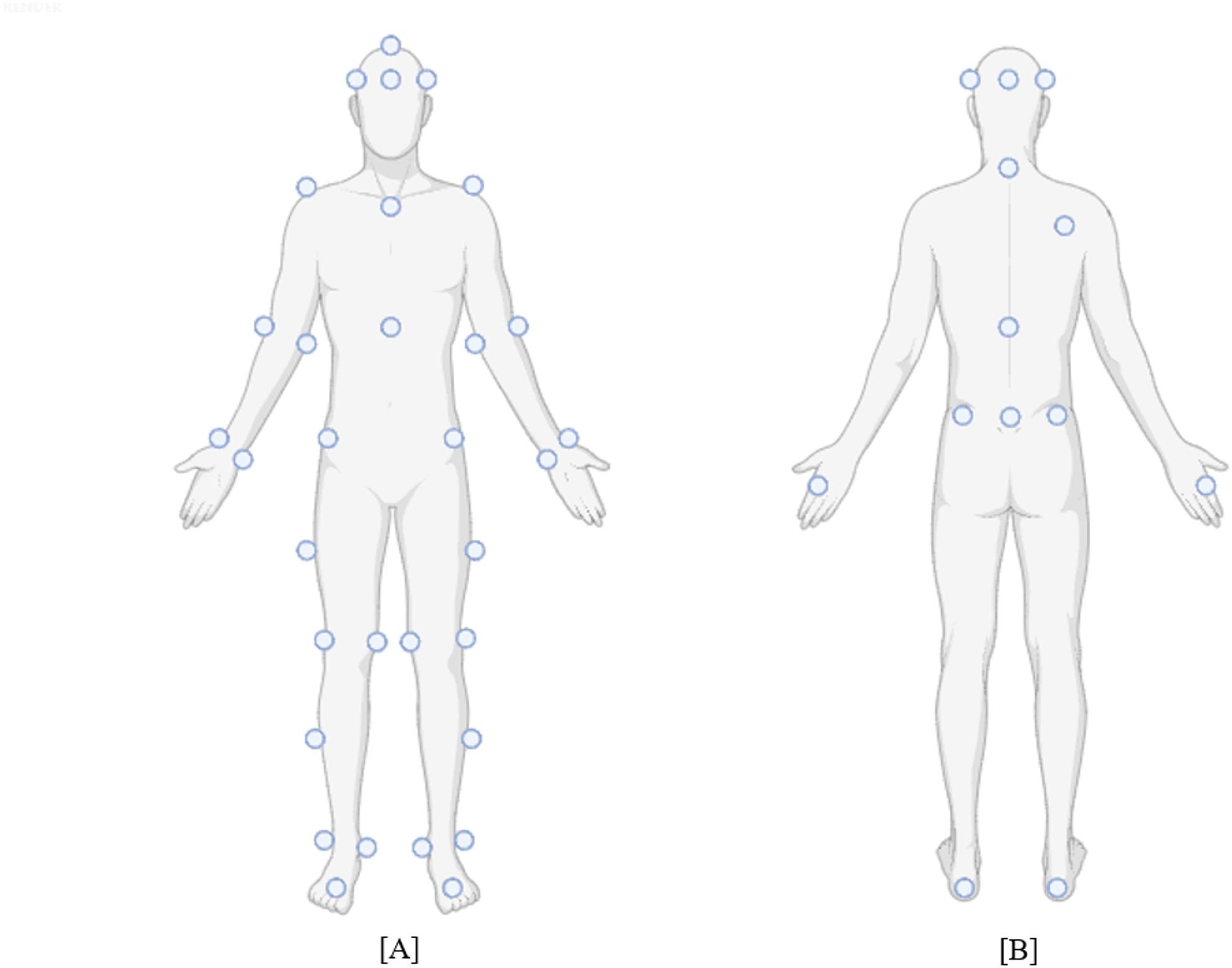
Helen-hayes marker set. **A** Anterior view, (**B**) Posterior view; In posterior view, markers overlapped with anterior view were omitted

Grading of the FAC classification was performed by physical therapist researchers who had passed the reliability test of the corresponding rehabilitation center. Participants wore short-sleeved shirts and shorts to prevent markers slipping caused by clothing, and infra-red reflective markers [Helen-Hayes marker set, 45 markers (Qualisys, Sweden), Fig. 2] were attached to their bodies. Subsequently, they were instructed to walk along a seven-meter path twice—once for the main data and once for preliminary data in case the main data was corrupted. The participants maintained their comfortable gait speed and walked straight ahead. Before the main experiment, two practice gait tests were conducted. The researcher was positioned nearby to prevent falls during walking, and the participants were allowed to rest for three to five minutes between trials to avoid fatigue. Additionally, a conventional motor evaluation was conducted to obtain functional scores after the 7 m waling trial.

### Functional evaluation

The participants underwent functional evaluations of gross motor ability including the Medical Research Council (MRC, Supplementary Table S2) grade for muscle strength and Passive Range of Motion (PROM) in the lower extremities, Motricity Index (MI), Berg Balance Scale (BBS), Trunk Impairment Scale (TIS), Motor Assessment Scale (MAS) and Timed Up and Go (TUG) test.

A licensed physical therapist researcher determined the scores. Before participating in this study, the evaluator conducted a preliminary imaging test on 30 or more participants with stroke according to the protocol of the clinical research team, and intra-rater and inter-rater reliabilities (internal reliability and mutual reliability with other therapists at CHA Bundang Medical Center) were established with correlation coefficients ≥ 0.95.

#### Medical research council

MRC is used to assess muscle strength in individual joints through manual evaluation. Eight bilateral muscle groups in the lower extremities (hip flexors, hip extensors, hip abductors, hip adductors, knee flexors, knee extensors, ankle dorsiflexors, and ankle plantarflexors) were measured. The grading system is categorized into six levels: Normal, Good, Fair, Poor, Trace, and Zero [25]. The grades from Good to Poor are further subdivided with “+” and “–” modifiers to provide a more precise assessment. In this study, MRC grades were converted into numerical scores (0–100) based on the CHA Bundang Medical Center standards [26, 27]. The detailed score distribution is presented in Table S2.

#### Passive range of motion

PROM, a test used to evaluate the extent of joint movement, is achieved without active muscle contraction. The degree of movement was generally determined using goniometers, and the participants were compelled to perform their single motion maximally [28]. In this study, measurements were taken for the following lower extremity movements: hip flexion, hip extension, hip abduction, hip adduction, hip external rotation, hip internal rotation, knee flexion, knee extension, ankle dorsiflexion, and ankle plantar flexion.

#### Motricity index

MI is utilized to assess muscle strength in the upper and lower limbs. However, in this study, only the muscle strength of the lower limb joints (hip, knee, and ankle) was evaluated. Each joint was assigned a score ranging from 0 to 33, with the total score calculated as the sum of the three individual scores plus one, resulting in a maximum possible score of 100 [29].

#### Berg balance scale

BBS was designed to evaluate both static and dynamic balance. It consists of 14 tasks, each scored on a scale from 0 to 4, with a total possible score ranging from 0 to 56 points. Scores between 41 and 56 indicate the ability to walk independently, scores between 21 and 40 suggest a dependent gait requiring assistance, and scores below 21 signify a high risk of falling during ambulation [30].

#### Trunk impairment scale

TIS is employed to assess an individual’s static sitting balance, dynamic sitting balance, and trunk coordination [31]. The subscales comprise multiple components, each containing three to ten evaluation criteria. The scores for each subscale could be graded 0 ~ 7, 0 ~ 10, and 0 ~ 6. The total score ranges from 0 to 23, providing an overall measure of trunk function.

#### Motor assessment scale

MAS is a tool used to evaluate various motor functions, including upper arm movements, hand coordination, walking, and the transition from sitting to standing. The assessment comprises eight components, each scored on a scale from 0 to 6 [32]. The total score ranges from a minimum of 0 to a maximum of 48, reflecting overall motor performance.

#### Timed-up-and-go test

TUG is utilized to assess functional abilities relevant to daily activities, including walking and the transition from sitting to standing. Participants begin in a seated position and, upon receiving a start signal from the evaluator, are required to walk a three-meter distance, turn around, and return to the starting point to sit down. The time taken to complete the task, measured from the start signal to the moment they resume the seated position, is recorded. Threshold values for identifying fall risk range from 10 to 33 s [14].

### Data processing

Kinematic and kinetic data were collected and synchronized using a data acquisition system (DAQ, 5695B). Inverse dynamics analyses were performed using Qualisys Track Manager and Visual3D. MATLAB (R2022a; MathWorks Inc., Natick, MA, USA) was used to extract time-series data for one complete gait cycle, defined as the interval from heel strike to the subsequent heel strike, for each participant. The data were filtered using a low-pass Butterworth filter with a cutoff frequency of 6 Hz, which is commonly applied in gait analysis to remove high-frequency noise while preserving the relatively slow components of human movement. Spatio-temporal data included proportion (%) of the stance phase, the state of the tracked lower extremity foot on the ground surface, and swing phase, the state of the tracked lower extremity foot not on the ground surface, during the gait cycle on both the affected and unaffected sides, and cadence, defined as the number of steps per minute. Peak values in the anterior, posterior, and superior directions of the ground reaction force (GRF, unit: N/kg) were derived. Among the biomechanical indices of the lower limb joints (hip, knee, ankle), kinetic data provided peak values for the sagittal plane moment (unit: Nm/kg) and power (unit: Nm/s/kg), while kinematic data provided maximum joint angle (unit: ˚) and ROM (unit: ˚). Among the units of each indicator, the notation “/kg” indicates normalization to body weight. Power was calculated as the product of moment and angular velocity. When the directions of moment and angular velocity were aligned, the result was defined as generation power; when they were opposite, it was defined as absorption power. In this study, the (+, -) sign in angle, moment, and power indicates the direction of movement of the segment or power mechanisms; therefore, the maximum or peak values represent the absolute maximum values considering the sign. Conventional evaluation data were recorded using Microsoft Excel software (Microsoft Office Professional Plus 2019; Microsoft Inc., Redmond, United States).

### Statistical analysis

All statistical analyses were performed using SPSS version 25.0 (IBM Corp., Armonk, NY, USA). Descriptive statistics are presented as mean ± standard deviation. The normality of each variable within the FAC 3, FAC 4, and healthy groups was assessed using the Shapiro–Wilk test. For variables that met the assumption of normality, one-way analysis of variance (ANOVA) was performed to examine group differences, followed by independent t-tests for post-hoc pairwise comparisons. For variables violating the normality assumption in any group, the Kruskal–Wallis test was used, and when significant, Mann–Whitney tests were conducted for post hoc comparisons. Spatiotemporal gait parameters, which typically show non-normal distributions in clinical populations, were also analyzed using Kruskal–Wallis and Mann–Whitney tests. Considering the limited sample size and exploratory nature of this study, no correction for multiple comparisons was applied. Instead, results were interpreted with caution, emphasizing the magnitude and consistency of between-group differences rather than isolated p-values. This approach has been adopted in several biomechanical and exploratory rehabilitation studies with small sample sizes, where overly conservative corrections could mask potentially meaningful trends [33, 34]. A significance level of *p* < 0.05 was considered statistically significant for all analyses.

## Results

### General participant characteristics classified by groups

Twelve patients with chronic stroke and six healthy individuals (four men, mean ± SD: age 65.17 ± 3.97 years, height 163.02 ± 11.15 cm, weight 67.25 ± 14.98 kg) were recruited and included in the final analysis comparing biomechanical parameters and functional measurement results. Based on gait ability, the patients were classified into either the FAC 3 group (N.=6, four men, mean ± SD: age 66.33 ± 5.24 years, height 163.33 ± 8.98 cm, weight 62.58 ± 10.13 kg, duration after onset 1220.67 ± 863.66 days) or the FAC 4 group (N.=6, four men, mean ± SD: age 68.33 ± 9.95 years, height 162.5 ± 7.04 cm, weight 61.52 ± 8.34 kg, duration after onset 1316.00 ± 596.80 days). Among the patients, eleven had supratentorial strokes (six in the FAC 3 group) and eight had infarctions (three in the FAC 3 group; Supplementary Table S1). There was no significant difference (*p* = 0.728; 0.988; 0.663) between the three groups in age, height, and weight. Furthermore, no demographic differences (*p* = 0.828), including duration after onset, were found between the FAC 3 and 4 groups (Table 1).

### The affected side

In the comparison of spatio-temporal parameters among the three groups, there was no significant difference (*p* > 0.05) in the affected lower limb (Table 2). In the comparison of kinematic indices, the patients with stroke, including both FAC 3 and 4 groups, showed significantly lower ROM values with reduced flexion angle in both hip and knee joints and reduced ankle plantar-flexion angles compared to the healthy group (*p* < 0.05), while no differences were observed between the two stroke groups (Table 3). In addition, the FAC 3 group showed lower ROM with a reduced range in the ankle joint than the healthy group (*p* = 0.021), with no differences between the FAC 4 and healthy groups.

**Table 2:** Comparisons of spatio-temporal indicators between the whole groups

Side	Parameters	Group			*p*-values		
		FAC 3 (*N*.=6)	FAC 4 (*N*.=6)	Healthy (*N*.=6)	FAC 3 vs. FAC 4	FAC 3 vs. Healthy	FAC 4 vs. Healthy
Affected side	Stance phase (%)	65.3 ± 7.8	64.9 ± 4.3	60.6 ± 2.0	0.818	0.394	***0.041*** †
	Swing phase (%)	34.7 ± 7.8	35.1 ± 4.3	39.5 ± 2.0	0.818	0.394	***0.041*** †
Unaffected side	Stance phase (%) **	76.4 ± 8.2	73.8 ± 7.9	60.6 ± 2.0	0.589	***0.002*** ††	***0.002*** ††
	Swing phase (%) **	23.6 ± 8.2	26.2 ± 7.9	39.5 ± 2.0	0.589	***0.002*** ††	***0.002*** ††
Cadence (steps/minute) **		42.0 ± 7.1	40.3 ± 7.5	53.5 ± 2.9	0.699	***0.004*** ††	***0.009*** ††
Gait speed (meter/second)		0.4 ± 0.3	0.4 ± 0.2	1.0 ± 0.1	0.818	***0.002*** ††	***0.002*** ††

*FAC* Functional Ambulation Category. Continuous values are presented as mean±standard deviation

**p<0.01 by Kruskal-Wallis test for analyzing differences between three groups

†*p*<0.05, ††*p*< 0.01 by Mann-Whitney test for Post-hoc analysis

**Table 3:** Comparisons of kinematic indicators between the whole groups

Parameters	Joint	Side	Motion	FAC 3	FAC 4	Healthy	FAC 3 vs. FAC 4			FAC 3 vs. Healthy			FAC 4 vs. Healthy		
				(N.=6)	(N.=6)	(N.=6)	*p-*values	95% CI	95% CI	*p-*values	95% CI	95% CI	*p-*values	95% CI	95% CI
								lower	upper		lower	upper		lower	upper
Maximum joints angle (˚) & ROM (˚)	Hip	Affected side	Flexion (+) **	16.89	17.11	32.53	0.964	−11.04	10.59	***0.001*** **††**	***−22.62***	***−8.66***	***0.009*** **††**	***−25.42***	***−5.41***
			Extension (-)	−4.79	−8.55	−10.92	0.403	−5.82	13.33	0.073	−0.68	12.93	0.517	−5.48	10.22
			ROM (˚) **	21.68	25.66	43.44	0.532	−17.7	9.74	***0.001*** **††**	***−31.56***	***−11.97***	***0.013*** **†**	***−30.29***	***−5.27***
		Unaffected side	Flexion (+) **	22.53	28.11	32.53	0.13	−13.11	1.95	***0.011*** **†**	***−16.82***	***−3.17***	0.101	***−9.86***	***1.03***
			Extension (-) *	−5.97	−6.21	−10.92	0.908	−4.33	4.82	***0.021*** **†**	***0.91***	***8.98***	***0.045*** **†**	0.13	9.28
			ROM (˚) ***	28.5	34.32	43.44	***0.047*** **†**	***−11.56***	***−0.09***	***< 0.001*** **†††**	***−20.51***	***−9.38***	***0.004*** **††**	***−14.56***	***−3.68***
	Knee	Affected side	Flexion (-) **	−30.64	−33.09	−63.41	0.783	−16.82	21.72	***< 0.001*** **†††**	***18.83***	***46.7***	***0.004*** **††**	***13.52***	***47.1***
			Extension (+)	1.17	3.15	−2.26	0.54	−8.92	4.97	0.341	−4.23	11.1	0.016*	***1.27***	***9.55***
			ROM (˚) **	31.82	36.24	61.15	0.532	−19.66	10.81	***< 0.001*** **†††**	***−40.47***	***−18.19***	***0.004*** **††**	***−39.67***	***−10.14***
		Unaffected side	Flexion (-) **	−43.84	−55.7	−63.41	***0.026*** **†**	***1.71***	***22.02***	***0.003*** **††**	***8.54***	***30.6***	***0.032*** **†**	***0.82***	***14.59***
			Extension (+) *	−4.42	−8.52	−2.26	0.274	−3.78	11.96	0.532	−9.61	5.29	***0.038*** **†**	***−12.09***	***−0.42***
			ROM (˚) ***	39.41	47.19	61.15	0.093	−17.21	1.66	***< 0.001*** **†††**	***−30.06***	***−13.41***	***0.018*** **†**	***−24.93***	***−2.99***
	Ankle	Affected side	Dorsiflexion (+)	7.9	7.6	9.03	0.903	−5.17	5.78	0.538	−5.06	2.81	0.574	−6.92	4.06
			Plantarflexion (-) *	−8.02	−12.97	−19.8	0.239	−3.86	13.75	***0.019*** **†**	***2.42***	***21.13***	***0.049*** **†**	***0.02***	***13.64***
			ROM (˚) *	15.92	20.56	28.82	0.292	−13.93	4.65	***0.021*** **†**	***−23.37***	***−2.43***	0.09	−18.06	1.54
		Unaffected side	Dorsiflexion (+)	9.04	12.68	9.03	0.155	−8.92	1.64	0.995	−4.6	4.62	0.115	−1.06	8.37
			Plantarflexion (-) ***	−8.06	−6.96	−19.8	0.735	−8.13	5.92	***0.002*** **††**	***5.27***	***18.21***	***0.005*** **††**	***4.85***	***20.84***
			ROM (˚) ***	17.1	19.64	28.82	0.308	−7.81	2.73	***0.013*** **†**	***−20.43***	***−3.03***	***0.036*** **†**	***−17.64***	***−0.74***

*FAC* Functional ambulation category, *ROM* Range of motion, *CI* Confidence Intervals

*p<0.05, **p<0.01, ***p<0.001 by ANOVA test for analyzing differences between three groups

†*p*<0.05, ††*p*<0.01, †††*p*<0.001 by independent t-test for post-hoc analysis

Differences in the kinetic indices of the affected limb mostly appeared between the stroke group and the healthy group (Table 4.). The peak joint moments were significantly reduced (*p* < 0.05) not only in hip flexion and extension, but also in ankle dorsiflexion and plantar flexion in patients with stroke. Between the FAC 3 and 4 groups, a difference appeared only in hip flexion, with a lower moment in the FAC 3 group (*p* = 0.044). The peak joint powers were reduced during generation and absorption of the hip and ankle joints and absorption at the knee joint on the affected side in patients with stroke (*p* < 0.05), while no significant differences were observed between the two stroke groups. Ground reaction forces in all directions were also reduced in patients with stroke compared to healthy individuals (*p* < 0.05), with no significant differences between the FAC 3 and 4 groups.

**Table 4.**
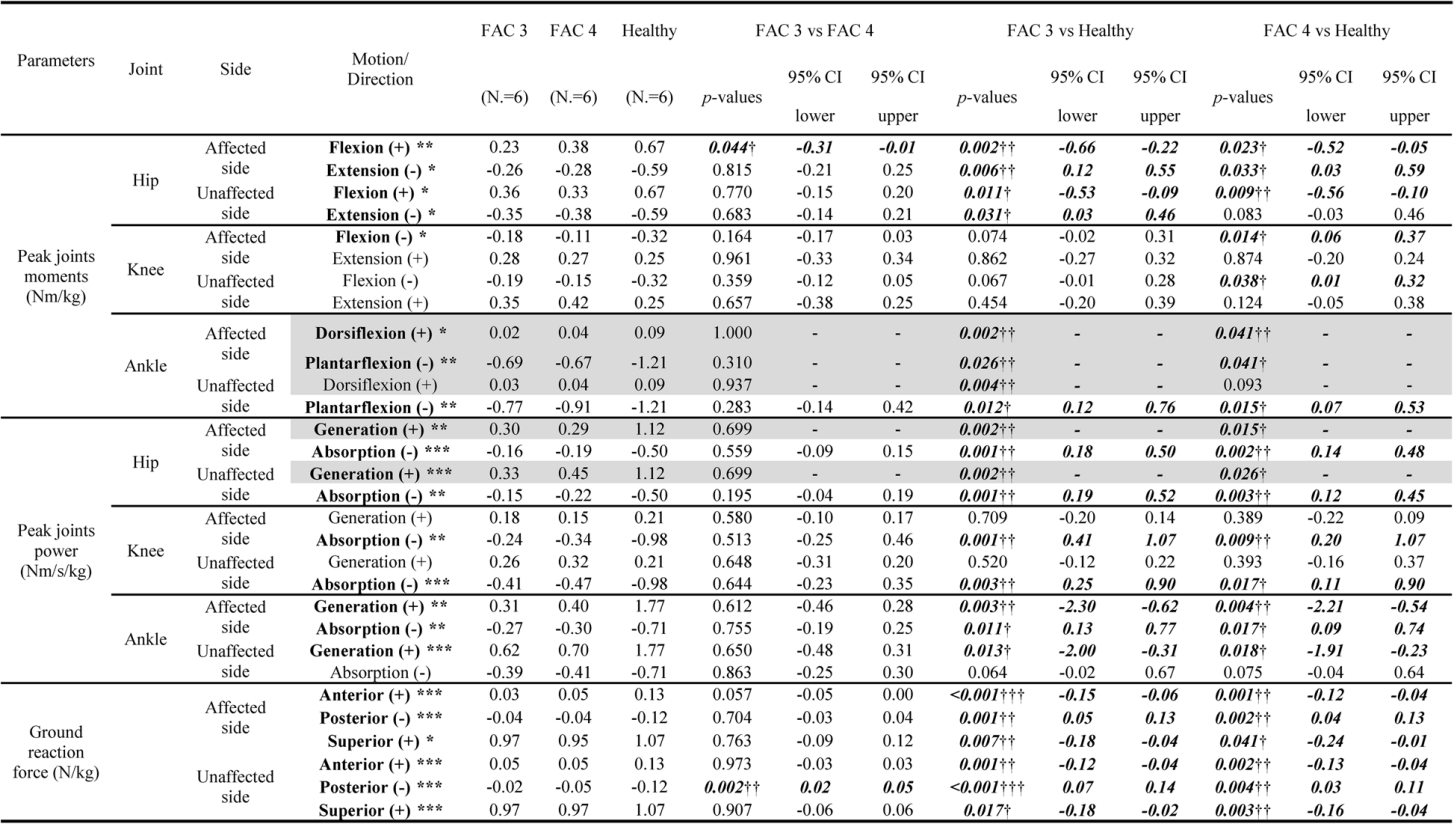
Comparisons of kinetic indicators between the whole groups

*FAC* Functional Ambulation category, *CI* Confidence Intervals. *p<0.05, **p<0.01, ***p<0.001 by Kruskal-Wallis or ANOVA tests for analyzing differences between three groups; †*p*<0.05, ††*p*<0.01, †††*p*<0.001 by Mann-Whitney or independent t-tests for post-hoc analysis; Grey shaded areas mean parameters analyzed by Kruskal-Wallis and Mann-Whitney tests, the others were analyzed by ANOVA and independent t-tests. The values of 95% CI were expressed in the results only from independent t-test

**Fig. 3:**
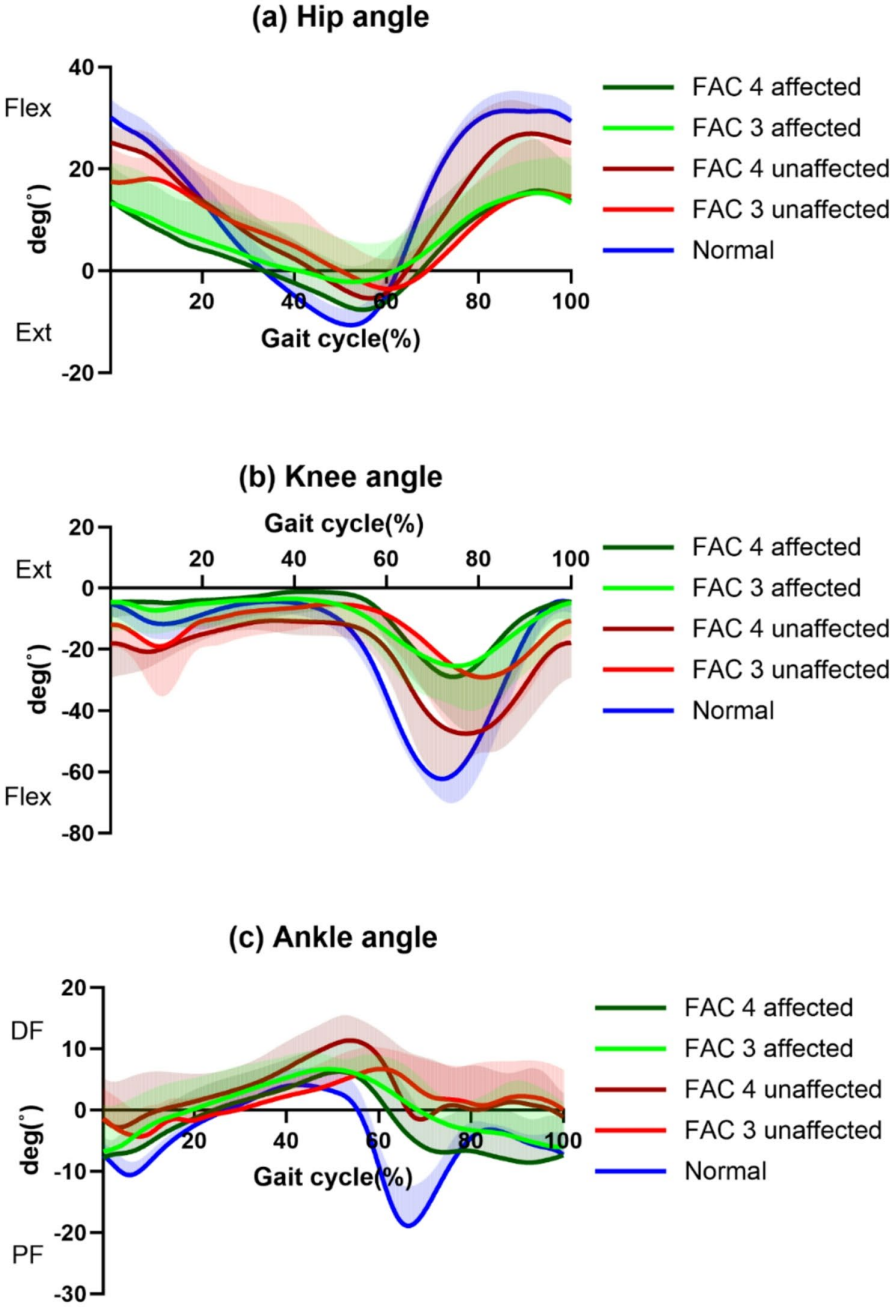
Angle trajectories of stroke groups and healthy adults in lower limb joints during the gait cycle. Bold lines represent the mean value of each group, and shaded areas show the positive or negative standard deviation. Shaded areas for the other standard deviation were eliminated for better clarity. *deg* degree *Flex* Flexion, *Ext* Extension, *DF* Dorsi-flexion, *PF* Plantar-flexion, *FAC* Functional Ambulation Category

**Fig. 4:**
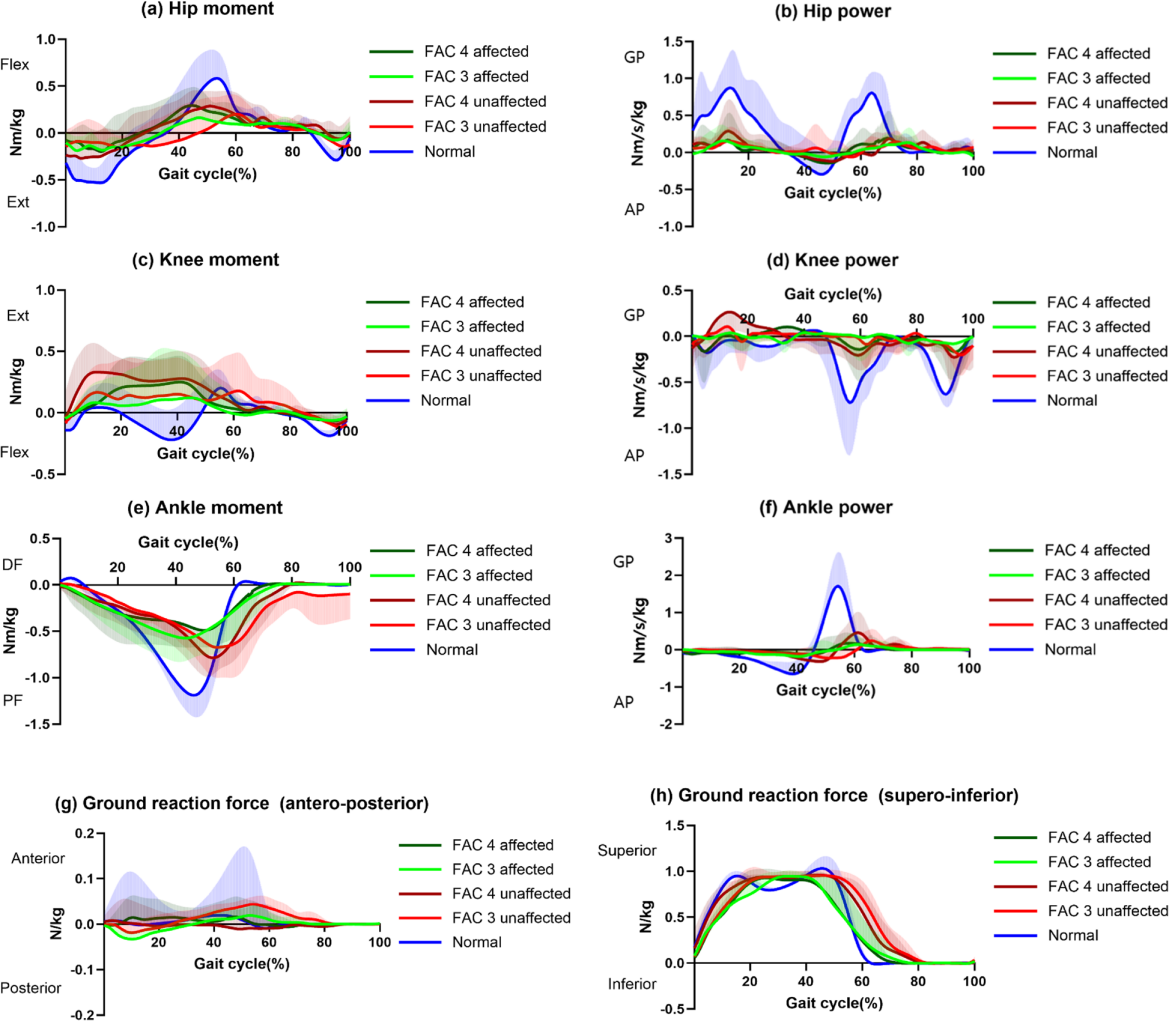
Kinetic parameters changes of stroke groups and healthy adults in lower limb joints during the gait cycle. Bold lines represent the mean value of each group, and shaded areas show the positive or negative standard deviation. Shaded areas for the other standard deviation were eliminated for better clarity. *Flex* Flexion, *Ext* Extension, *DF* Dorsiflexion, *PF* Plantarflexion, *GP* Generation Power, *AP* Absorption Power, *FAC* Functional Ambulation Category

The trajectory of each joint angle in all groups during the gait cycle is illustrated in Fig. 3. The changes in each kinetic parameter during the gait cycle are shown in Fig. 4.

### The unaffected side

In comparison of spatiotemporal parameters, patients with stroke, including both FAC 3 and FAC 4 groups, showed a shorter swing phase duration (and a correspondingly longer stance phase duration) on the unaffected side (*p* < 0.01) and lower cadences (*p* < 0.01) compared to the healthy group (Table 2). Regarding kinematic indices, the stroke groups showed significantly lower (*p* < 0.05) values than the healthy group in hip ROM, hip flexion angle, knee ROM, ankle plantarflexion angle, and ankle ROM (Table 3). Between the FAC 3 and FAC 4 groups, a significant difference was observed only in hip ROM, with a lower range in the FAC 3 group (*p* = 0.047). The FAC 3 group showed a smaller range of hip extension angle compared to the healthy group (*p* = 0.011), while no significant difference was found between the FAC 4 group and the healthy group.

The kinetic indices revealed significantly lower (*p* < 0.05) peak joint moments of hip flexion and ankle plantarflexion in both stroke groups compared to the healthy group (Table 4.). In addition, the FAC 3 group showed lower moments of hip extension and ankle dorsiflexion than the healthy group (*p* < 0.05). Peak joint power was reduced during hip generation and absorption, knee absorption, and ankle generation in patients with stroke (*p* < 0.05), with no significant differences between the two stroke groups. The GRFs in all directions were also significantly reduced in patients with stroke compared to the healthy group (*p* < 0.05). Additionally, posterior GRF was lower in the FAC 3 group than in the FAC 4 group (*p* = 0.002).

### Functional measurements

The clinical evaluation included MRC, PROM, MI, BBS, TIS, MAS, and TUG (Table 5). Only the results pertaining to the lower limb joints, as reported in this study, were analyzed for MRC, ROM, and MI. Only the BBS was lower in the FAC 3 group (43.571 ± 4.158 points; mean ± SD) than in the FAC 4 group (48.143 ± 5.146 points; mean ± SD; *p* = 0.041), while the scores of the other measures were not significantly different between the stroke groups.

**Table 5:** Comparison of functional measurements between FAC 3 and FAC 4 group

Evaluation	Side	Index	FAC 3 group Mean ± SD	FAC 4 group Mean ± SD	*p*-values
MRC^a^ (points)	Unaffected side	Hip flexion	72.857 ± 7.559	78.571 ± 3.780	0.310
		Hip extension	72.857 ± 4.880	75.714 ± 7.868	0.589
		Hip abduction	64.286 ± 13.973	75.714 ± 7.868	0.310
		Hip adduction	68.571 ± 10.690	75.714 ± 7.868	0.310
		Knee flexion	74.286 ± 5.345	78.571 ± 3.780	0.394
		Knee extension	75.714 ± 5.345	78.571 ± 3.780	0.699
		Ankle dorsi-flexion	75.714 ± 5.345	78.571 ± 3.780	0.699
		Ankle plantar-flexion	70.000 ± 10.000	78.571 ± 3.780	0.132
		Lower extremities (total)	574.286 ± 51.594	620.000 ± 41.231	0.310
	Affected side	Hip flexion	60.000 ± 8.165	60.714 ± 15.924	0.818
		Hip extension	45.714 ± 14.268	53.571 ± 15.999	0.485
		Hip abduction	50.714 ± 13.671	53.571 ± 14.920	0.699
		Hip adduction	50.714 ± 13.671	53.571 ± 12.488	0.699
		Knee flexion	53.571 ± 16.511	54.286 ± 20.089	0.937
		Knee extension	60.714 ± 17.423	63.571 ± 15.469	0.937
		Ankle dorsi-flexion	32.857 ± 32.514	38.571 ± 29.114	0.818
		Ankle plantar-flexion	30.000 ± 28.805	47.143 ± 25.472	0.310
		Lower extremities (total)	384.286 ± 114.834	425.000 ± 128.809	0.699
PROM^b^ (degree)	Unaffected side	Hip flexion	100.000 ± 0.000	100.000 ± 0.000	1.000
		Hip extension	10.000 ± 0.000	10.000 ± 0.000	1.000
		Hip abduction	24.286 ± 1.890	25.000 ± 0.000	1.000
		Hip adduction	15.000 ± 0.000	15.000 ± 0.000	1.000
		Hip external rotation	29.714 ± 0.756	30.000 ± 0.000	0.699
		Hip internal rotation	20.000 ± 0.000	20.000 ± 0.000	0.699
		Knee flexion	110.000 ± 0.000	110.000 ± 0.000	1.000
		Knee extension	0.000 ± 0.000	0.000 ± 0.000	1.000
		Ankle dorsi-flexion	10.000 ± 0.000	10.000 ± 0.000	1.000
		Ankle plantar-flexion	20.000 ± 0.000	20.000 ± 0.000	1.000
		Lower extremities (total)	938.570 ± 20.165	941.429 ± 12.150	0.818
	Affected side	Hip flexion	100.000 ± 0.000	100.000 ± 0.000	1.000
		Hip extension	10.000 ± 0.000	10.000 ± 0.000	1.000
		Hip abduction	24.286 ± 1.890	25.000 ± 0.000	1.000
		Hip adduction	15.000 ± 0.000	15.000 ± 0.000	1.000
		Hip external rotation	29.429 ± 1.512	30.000 ± 0.000	0.699
		Hip internal rotation	20.000 ± 0.000	20.000 ± 0.000	0.699
		Knee flexion	110.000 ± 0.000	110.000 ± 0.000	1.000
		Knee extension	0.000 ± 0.000	0.000 ± 0.000	1.000
		Ankle dorsi-flexion	5.714 ± 7.868	7.143 ± 7.559	0.699
		Ankle plantar-flexion	20.000 ± 0.000	20.000 ± 0.000	1.000
		Lower extremities (total)	903.286 ± 70.616	937.714 ± 20.734	0.310
MI (points)	Unaffected side	Lower extremities (total)	95.429 ± 9.071	97.714 ± 6.047	0.699
	Affected side		65.429 ± 18.528	66.286 ± 16.540	0.937
BBS (points)		Total score	43.571 ± 4.158	48.143 ± 5.146	***0.041*** *
TIS (points)		Total score	13.286 ± 3.638	14.857 ± 2.340	0.589
MAS (points)		Total score	31.000 ± 9.018	38.714 ± 7.973	0.180
TUG (seconds)		Run-time	28.373 ± 11.332	19.506 ± 6.361	0.310

*SD* Standard Deviation, *MRC* Medical Research Council, *PROM* Passive Range of Motion, *MI* Motricity Index, *BBS* Berg Balance Scale, *TIS* Trunk Impairment Scale, *MAS* Motor Assessment Scale, *TUG* Timed-Up and Go test

*p<0.05 by Mann-Whitney test

MRC graded-based score distribution was presented in Table S2

## Discussion

This study compared the biomechanical characteristics of gait between patients with stroke classified as FAC 3 and FAC 4 and healthy adults. In the clinical field, FAC 3 and FAC 4 levels could mark a critical threshold between dependent and independent walking, reflecting substantial differences in balance control, motor coordination, and compensatory gait strategies. Hence, the authors believe that biomechanical parameter differences through instrumental gait analysis across these FAC levels would provide mechanistic insights for developing individualized gait rehabilitation strategies and found some significant parameters.

Between FAC 3 and 4 groups, the posterior GRF at initial contact on the unaffected side emerged as a significant differentiator [35]. The posterior GRF, indicative of the loading response and propulsion at the onset of the stance phase, was particularly greater in the FAC 4 group, suggesting gait mechanics more akin to those of healthy individuals [36]. Stroke survivors frequently exhibit impairments in both the affected and contralateral “unaffected” extremities due to inadequate weight-bearing, proprioceptive disturbances, or disuse-related immobilization [37, 38]. Consequently, gait patterns may become bilaterally abnormal from a biomechanical perspective. Our results also identified critical kinetic variables associated with initial contact, specifically the peak ankle dorsiflexion moment and the peak hip extension moment on the unaffected side. The peak ankle dorsiflexion moment, primarily reflecting the eccentric activation of dorsiflexors such as the tibialis anterior [39, 40], was reduced in the FAC 3 group than in healthy group, whereas the FAC 4 group did not exhibit this deficit. The reduction in dorsiflexor control may account for the diminished posterior GRF in the FAC 3 group, indicating insufficient shock absorption and propulsion during early stance. Eccentric contraction training for ankle dorsiflexors including tibialis anterior could be demanded for stroke patients with FAC 3 and the training could benefit on improving the initial contact during the gait.

Similarly, the peak hip extension moment on the unaffected side, reflecting the concentric contraction of hip extensors, was greater in the FAC 4 group, suggesting superior neuromuscular function. Notably, this hip extension moment temporally coincided with the peak hip flexion moment on the affected side during pre-swing, which reflects eccentric contraction of the hip flexors [39]. The FAC 4 group exhibited significantly higher peak hip flexion moments on the affected side compared to the FAC 3 group, approaching values observed in healthy groups. Given that eccentric contractions are generally better preserved than concentric contractions following a stroke [41, 42], these findings suggest that strengthening eccentric hip flexors on the affected side may indirectly enhance concentric hip extensor performance on the unaffected side, thereby contributing to improved posterior GRF. Beyond kinetic differences, significant kinematic differences also existed between the FAC 3 and FAC 4 groups, particularly in the hip and knee joints of the unaffected side. The FAC 4 group demonstrated greater hip ROM, supporting better swing-phase control and indicating a more advanced stage of functional recovery. Additionally, higher peak knee flexion angles in the FAC 4 group suggest more coordinated interjoint dynamics during swing. These findings align with previous studies indicating that proximal joints (e.g., hips) tend to recover more quickly than distal joints (e.g., ankles) following neurological injury [43–45]. Considering the similar performance of the PROM between the FAC 3 and 4 groups, education motivating the larger hip and knee movement during gait could improve the gait pattern of the FAC 3 group.

In this study, the FAC 4 group showed various biomechanically superior parameters compared to the FAC 3 group; however, there were also many parameters that from both groups were inferior to healthy individuals. This suggests that even in stroke groups where walking was possible to some extent, there are still areas that need improvement. In comparison to healthy individuals, both stroke groups exhibited impairments in spatio-temporal parameters including reduced swing phase duration on the unaffected side, lower cadence, and slow gait speed. Kinematic differences included reduced ROM in both the affected and unaffected lower extremities of the stroke groups, except for the affected ankle. Kinetic results demonstrated significantly decreased joint absorption power across both lower extremities, excluding the unaffected ankle.

In terms of spatio-temporal gait parameters, patients with stroke demonstrated a reduced swing phase proportion on the unaffected side compared to the healthy group, possibly due to difficulties maintaining single-limb support on the affected side [46]. Furthermore, both stroke groups showed reduced cadence compared to the healthy group; however, no significant difference in the swing phase proportion on the affected side was observed. These findings could indicate persistent asymmetrical and abnormal gait patterns [47]. The stroke groups also showed slow gait speed compared to the healthy group, could influence the abnormal gait patterns [48]. These findings underscore the necessity for targeted interventions that focus on improving gait speed and symmetry in post-stroke rehabilitation. Furthermore, this study showed persistent gait dysfunction in the FAC4 group despite independent ambulation. Tailored rehabilitation strategies, including rhythmic auditory stimulation or treadmill training, an evidence-based approach for enhancing spatiotemporal gait parameters, may still be required to address the remaining dysfunction [49–51]. In addition, both stroke groups exhibited significantly reduced ROM compared to healthy adults, particularly in hip and knee flexion and ankle plantar-flexion, which are crucial for achieving adequate stride length. These results reflect limitations in dynamic joint mobility, suggesting the need for flexibility and mobility training across joints in chronic stroke rehabilitation. Additionally, neither stroke group achieved a neutral ankle position during the swing phase (Fig. 3-c), indicating the presence of a persistent drop-foot pattern, which is likely attributable to a diminished dorsiflexor moment that increases the risk of tripping or falling, particularly on the affected side [41]. Notably, such gait deviations may not be detected by conventional clinical tools such as the MRC scale. These findings may suggest functional deficits, including coordination of ankle muscles, in patients with chronic stroke despite sufficient ROM and muscle strength when measured in a resting position. The presence of drop foot highlights deficits in distal motor control, a commonly reported issue in adults with chronic stroke [52, 53]. In terms of joint kinetics, both stroke groups exhibited diminished absorption power, an essential shock-absorbing mechanism, compared to the healthy group across all joints except the unaffected ankle. This kinetic deficiency supports the necessity to implement rehabilitation protocols that emphasize eccentric strength training and coordination between joint mobility and muscle function to promote safer and more efficient gait patterns.

Among the standard clinical assessment tools, only the BBS effectively distinguished between the two stroke groups. Other assessments failed to identify functional differences that were evident through biomechanical analyses, highlighting the limitations of conventional clinical measurements. Although BBS scores ranging from 41 to 56 are generally associated with independent ambulation [30], both stroke groups exhibited average scores within this range; however, biomechanical impairments persisted. This discrepancy suggests that even the BBS may lack the sensitivity to detect subtle but functionally significant gait deviations. In contrast, biomechanical variables, such as joint power and GRF profiles, emerged as more sensitive indicators of gait recovery, consistent with previous findings [22]. The results of this study underscore the importance of integrating biomechanical assessments into clinical evaluations to support individualized rehabilitation planning. Specifically, interventions targeting eccentric contractions in the proximal joints of the unaffected extremities may promote symmetrical gait mechanics. Furthermore, enhancing function in the affected extremity may help normalize performance on the unaffected side by reducing compensatory strategies. These findings emphasize the need for balanced, patient-specific rehabilitation approaches guided by each individual’s biomechanical profile.

Briefly, this study demonstrated that biomechanical analyses can identify subtle yet clinically meaningful gait differences between stroke survivors classified as FAC 3 and FAC 4, differences not fully captured by conventional clinical assessments. Kinetic variables, including posterior ground reaction force and ankle dorsiflexion moments on the unaffected side, as well as hip extension and flexion moments, have emerged as sensitive indicators of motor recovery and compensatory gait control. These findings suggest that integrating biomechanical parameters into clinical evaluations may enhance the precision of functional classification and support individualized rehabilitation planning. Nonetheless, because electromyography (EMG) data were not collected, mechanistic interpretations regarding muscle activation timing and neuromuscular control remain speculative. Additionally, correlation analysis between functional outcomes and gait parameters, and the correction for multiple comparisons had not been applied. Future studies with larger sample sizes, incorporation of EMG, additional statistical analysis and inclusion of upper-body joint analyses are warranted to validate these proposed mechanisms and to improve interpretability. If confirmed, the identified biomechanical markers may serve as objective targets for the development of personalized rehabilitation interventions, particularly those emphasizing eccentric control and interlimb coordination, to promote safer and more efficient gait recovery after stroke.

## Conclusion

This study investigated biomechanical indicators among patients with chronic stroke, including those with dependent gait (FAC level 3), and compared these responses to a group of age-matched healthy adults. Using motion analysis, we provided an alternative approach to assess gait conditions that are difficult to characterize through conventional evaluations and visual observation alone, presenting both kinematic and kinetic results of lower limbs on the sagittal plane. Results demonstrated differences in biomechanical indicators based on FAC levels, which may serve as foundational data for developing customized rehabilitation strategies tailored to individual gait conditions. Consequently, rehabilitation strategies targeting enhanced eccentric contraction—particularly in the proximal joints of the unaffected side —may be effective in helping patients with stroke walk on level surfaces.

## Supplementary Information

Supplementary Material 1.

## Data Availability

The data that support the findings of this study are available from the corresponding author upon reasonable request.

## Abbreviations

FAC: Functional Ambulation Category
PROM: Passive Range of Motion
MI: Motricity Index
BBS: Berg Balance Scale
TIS: Trunk Impairment Scale
MAS: Motor Assessment Scale
TUG: Timed Up and Go test
MRC: Medical Research Council
GRF: Ground Reaction Force
ROM: Range of Motion
IRB: Institutional Review Board
