# Supplementary material for: Comparison of gait parameters between patients with chronic stroke at different ambulation levels and healthy adults: a prospective observational study
Source: BMC Sports Sci Med Rehabil. 2025 Dec 1;18:9. doi: 10.1186/s13102-025-01444-4 (PMC12771758; doi:10.1186/s13102-025-01444-4)
Supplement: Supplementary file 1 — Supplementary Material 1. [file 13102_2025_1444_MOESM1_ESM.docx]

# Supplementary Material

Table S1. Individual demographic and pathological information of participants

| FAC grade | Subject No. | Age (years) | Height (cm) | Weight (kg) | Gender (male/female) | Onset duration (days) | Diagnosis | Unaffected side (right/left) |
| --- | --- | --- | --- | --- | --- | --- | --- | --- |
| 3 | 01 | 70 | 173.1 | 74.9 | male | 1760 | left frontotemporal lobe infarction | left |
| 3 | 02 | 65 | 171.9 | 72.9 | male | 1814 | infarctions in right internal carotid artery territory | right |
| 3 | 03 | 68 | 160.2 | 65 | female | 599 | left thalamic intracerebral hemorrhage | left |
| 3 | 04 | 57 | 160.1 | 59.3 | male | 2353 | left basal ganglia, intracerebral hemorrhage, intraventricular hemorrhage | left |
| 3 | 05 | 72 | 165.8 | 52.2 | male | 583 | right frontal lobe, temporal lobe to insula acute infarctions | right |
| 3 | 06 | 66 | 148.9 | 51.15 | female | 215 | right basal ganglia, intraventricular hemorrhage, intracerebral hemorrhage | right |
| 4 | 07 | 71 | 159.6 | 60.4 | male | 2255 | right middle cerebral artery infarction | right |
| 4 | 08 | 67 | 170 | 60 | male | 764 | left basal ganglia infarction | left |
| 4 | 09 | 53 | 163.6 | 56.4 | female | 1469 | left basal ganglia, intracerebral hemorrhage | left |
| 4 | 10 | 80 | 151.5 | 53.6 | female | 826 | Both cerebellum infarction (left severe) | right |
| 4 | 11 | 62 | 170 | 61.2 | male | 885 | right middle cerebral artery infarction | right |
| 4 | 12 | 77 | 160.3 | 77.5 | male | 1697 | multiple small subacute infarction scattered in left middle cerebral artery territory infarction, small infarctions in left basal ganglia caudate head with some microbleeds | left |
| 5 | 13 | 64 | 149.5 | 52.2 | female | - | - | - |
| 5 | 14 | 66 | 165.6 | 73.7 | male | - | - | - |
| 5 | 15 | 68 | 163.3 | 59.7 | male | - | - | - |
| 5 | 16 | 61 | 166.4 | 56 | male | - | - | - |
| 5 | 17 | 71 | 152.6 | 68.8 | female | - | - | - |
| 5 | 18 | 61 | 180.7 | 93.1 | male | - | - | - |

FAC, Functional Ambulation Category.

Table S2. Medical Research Council grade-based score distribution

| Grade | Score |
| --- | --- |
| Normal | 100 |
| Good + | 90 |
| Good | 80 |
| Good - | 70 |
| Fair + | 60 |
| Fair | 50 |
| Fair - | 40s |
| Poor + | 35 |
| Poor | 25 |
| Poor - | 15 |
| Trace | 5 |
| zero | 0 |

Scores were distributed by standards of CHA Bundang medical center, department of rehabilitation medicine.
